# RhBMP-2 Activates Hippo Signaling through RASSF1 in Esophageal Cancer Cells

**DOI:** 10.1038/srep26821

**Published:** 2016-05-27

**Authors:** Soo Mi Kim, Shuai Ye, So-Young Rah, Byung Hyun Park, Hongen Wang, Jung-Ryul Kim, Seung Ho Kim, Kyu Yun Jang, Kwang-Bok Lee

**Affiliations:** 1 Department of Physiology, Institute for Medical Sciences, Research Institute of Clinical Medicine of Chonbuk National University-Biomedical Research Institute of Chonbuk National University Hospital Chonbuk National University Medical School, Jeon Ju, 561-180, Republic of Korea; 2 Department of Orthopedic Surgery, Institute for Medical Sciences, Research Institute of Clinical Medicine of Chonbuk National University-Biomedical Research Institute of Chonbuk National University Hospital Chonbuk National University Medical School, Jeon Ju, 561-180, Republic of Korea; 3 Department of Biochemistry, Institute for Medical Sciences, Research Institute of Clinical Medicine of Chonbuk National University-Biomedical Research Institute of Chonbuk National University Hospital Chonbuk National University Medical School, Jeon Ju, 561-180, Republic of Korea; 4 Department of Gastroenterology, Institute for Medical Sciences, Research Institute of Clinical Medicine of Chonbuk National University-Biomedical Research Institute of Chonbuk National University Hospital Chonbuk National University Medical School, Jeon Ju, 561-180, Republic of Korea; 5 Department of Pathology, Institute for Medical Sciences, Research Institute of Clinical Medicine of Chonbuk National University-Biomedical Research Institute of Chonbuk National University Hospital Chonbuk National University Medical School, Jeon Ju, 561-180, Republic of Korea

## Abstract

Despite that recombinant human bone morphogenetic protein-2 (rhBMP-2) has been reported as a stimulatory effecter of cancer cell growth because of its characteristic like morphogen, the biological functions of rhBMP-2 in human esophageal cancer cells are unknown. The purpose of this study was to investigate whether rhBMP-2 has an inhibitory effect on the growth of human esophageal squamous carcinoma cells (ESCC). RhBMP-2 significantly inhibited proliferation of ESCC cells in a dose-dependent manner in the MTT assay. Cell cycle arrest at the G1 phase was induced 24 h after rhBMP2 treatment. RhBMP-2 also reduced cyclin D_1_, cyclin-dependent kinase (CDK) 4 and CDK 6 activities, and stimulated p-Smad1/5/8, p53, and p21 levels at 12 h. In contrast, rhBMP-2 diminished poly (ADP-ribose) polymerase (PARP) protein expression levels and activated cleaved PARP, cleaved caspase-7, and cleaved-caspase 9 levels in ESCC cells. In addition, rhBMP-2 increased MST1, MOB1, and p-YAP protein levels and the RASSF1 binds Mst1 more upon treatment with rhBMP2. The induced p-YAP expression in TE-8 and TE-12 cells by rhBMP-2 was reversed by the RASSF1 knockdown. *In vivo* study, rhBMP-2 decreased tumor volume following subcutaneous implantation and showed higher radiologic score (less bony destruction) after femoral implantation compared to those in a control group. These results suggest that rhBMP-2 inhibits rather than activates proliferation of human esophageal cancer cells which is mediated through activating the hippo signaling pathway.

## Introduction

Recombinant human bone morphogenetic protein-2 (rhBMP-2) has been used most commonly as a spine graft substitute since it was introduced commercially in 2002^1^,^2^,^3^. However, several safety issues including a possible cancer risk due to rhBMP-2 have been reported because both BMPs and their receptors have been found in human tumors^1^. Many researchers have reported that the use of rhBMP-2 in bone surgery is definitely related to a cancer risk, although they did not show incontrovertible evidence of the function of rhBMP-2 for promoting tumorigenesis or metastasis^4^. In contrast, a recent large cohort study revealed that administering rhBMP-2 at the time of spine surgery was not associated with cancer development^5^. The use of rhBMP-2 in bone surgery for cancer risk has been debated for a decade. In addition, a study using an oral carcinoma cell line showed that tumor xenografts established with rhBMP-2-treated cells induced more rapid local cancer growth that resulted in worse animal survival as compared to that in the control group^6^. A significant increase in tumor cell invasion due to rhBMP-2 treatment has been reported^7^. However, our recent published data show that rhBMP-2 has an anticancer effect *in vitro* and *in vivo* in breast cancer cell lines^8^. Despite continual efforts to understand the biological functions of rhBMP-2 in human tissues and cells, its safety remain largely unknown.

Because the increase of many genetic alterations drives cancer development, the Hippo pathway, which has been recently identified in *Drosophila*, has come under special scrutiny^9^,^10^,^11^. The Hippo pathway is an evolutionarily conserved regulator for the cell cycle, growth, and proliferation^12^. Because of overgrowth of cells and tissues when Hippo pathway components become mutated, the Hippo pathway has been postulated to be an important regulator of human cancer. The core Hippo pathway is a kinase cascade was consist of two Hipo homologs mammalian sterile twenty (Mst1 and Mst2), one Salvador protein (Sav) homolog (WW45 or Sav1), two Wts homologs large tumor suppressor (Lats1 and Lats2), and two Mats homologs (MOBKL1A and MOBKL1B, usually indicated to as Mob1) which behave as tumor suppressors^12^. Moreover, MST1/2 phosphorylates Sav, Lats1/2, and Mob. Lats1/2 phosphorylates Yes-associated protein (YAP). These genes inhibit the function of YAP, a key downstream effector of the Hippo pathway. Phosphorylated YAP accumulates in the cytoplasm and stimulates proteolysis. Hippo components are stimulated by other proteins including the upstream regulator of the Hippo pathway, ras association domain family (RASSF), a subgroup of Ras effector proteins^13^. It has reported RASSF1 promote apoptosis through regulate Mst1/2 activity^14^,^15^. Deregulation of the Hippo pathway has been reported many times in human carcinomas^12^,^16^,^17^,^18^. Although the Hippo pathway plays an important role in cell survival and proliferation, the relationship between the Hippo pathway and rhBMP-2 has not been addressed. Here, we investigated whether rhBMP-2 has an effect on the growth of human esophageal squamous carcinoma cells and whether rhBMP-2 regulates the Hippo signaling pathway in human esophageal squamous carcinoma cells. We demonstrated that rhBMP-2 inhibits *in vitro* proliferation of human esophageal squamous carcinoma cells by activating the Hippo pathway, and that it suppresses xenograft-implanted human esophageal tumors *in vivo*.

### Effect of rhBMP-2 on proliferation of TE-8 and TE-12 cells

**Figure 1:**
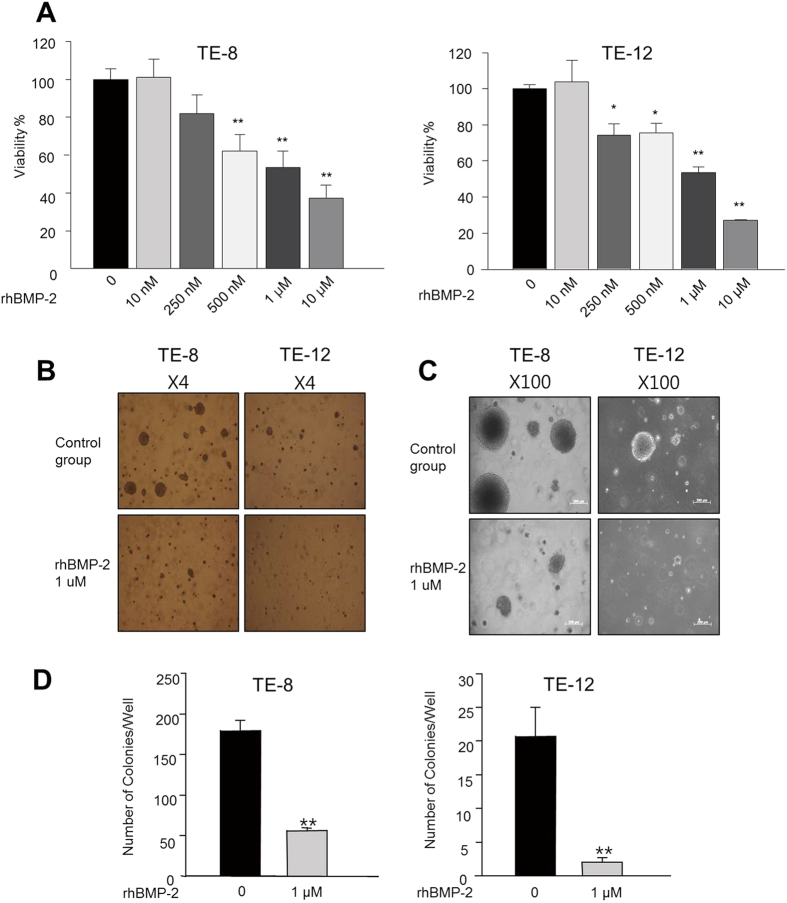
Effects of rhBMP-2 on TT, TE-8, and TE-12 cell proliferation and colony formation. rhBMP-2 inhibited cell proliferation in a dose-dependent manner. Consistent with the MTT assay, significantly fewer colonies formed compared with those in control cancer cells in the presence of 1 μM rhBMP-2 after 30 days (**B–D**). Each value represents mean ± standard error of at least three independent experiments with triplicate plates. ^*^P < 0.05, ^**^P < 0.01 vs. untreated cells.

The 3-(4,5-dimethylthiazol-2-yl)-2,5-diphenyltetrazolium bromide (MTT) assay was performed to determine the effects of rhBMP-2 on cytotoxicity in the TE-8 and TE-12 cell lines. As shown in Fig. 1, the TE-8 and TE-12 cell lines were treated in a dose-dependent manner with rhBMP-2 for 72 h. Treatment with rhBMP-2 (0, 0.01, 0.25, 0.5, 1, and 10 μM) resulted in inhibited cell proliferation in the TE-8 cell line (100.93 ± 9.90, 81.74 ± 10.10, 62.32 ± 8.39, 53.43 ± 3.21, and 37.31 ± 6.7%, respectively). rhBMP-2 inhibited cell proliferation in the TE-12 cell line in a dose dependent manner (0, 0.01, 0.25, 0.5, 1, 10 μM; 103.91 ± 11.90, 74.42 ± 6.26, 75.73 ± 5.13, 53.62 ± 8.89, 27.29 ± 0.42%, respectively). Consistent with the MTT assay, significantly fewer colonies formed compared with those in control cancer cells in the presence of 1 μM rhBMP-2 after 30 days (Fig. 1B–D).

### Induction of G1 arrest in TE-8 and TE-12 cells following rhBMP-2 treatment

**Figure 2:**
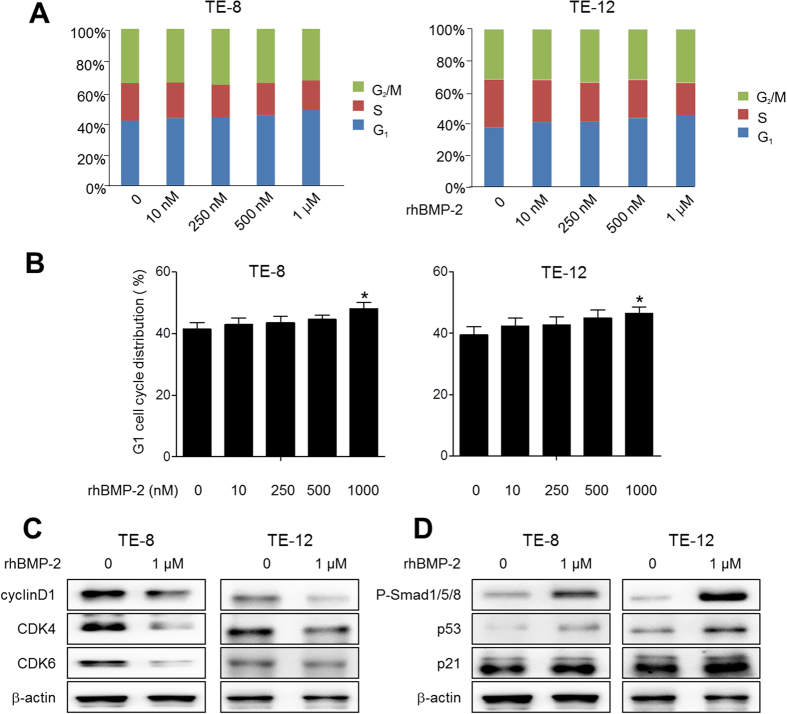
The effect of BMP-2 on cell cycle distribution in TE-8 and TE-12 cell lines. Treatment with rhBMP-2 was applied in a dose-dependent manner (0, 10, 250, 500, and 1000 nM) to TE-8 and TE-12 cell lines at 24 h. ^*^P < 0.05, ^**^P < 0.01 vs. untreated cells in G1 cell cycle distribution (**A**,**B**). Both cell lines were treated with 1 μM rhBMP-2 that induced both a decrease in G1 phase-related protein expression (e.g., cyclin D1, CDK 4, and CDK 6) as well as the increased expression of p-Smad 1/5/8, p53, and p21 (**C**,**D**). The gels have been run under the same experimental conditions. The full-length blots and the cropped blots are presented in Supplementary Figure 2. Data are a representative of three independent experiments. β**-**actin was used as an internal control.

Based on the cytotoxic response of esophageal cancer cells to rhBMP-2, cell cycle progression was analyzed by flow cytometry. As shown in Fig. 2A, the TE-8 and TE-12 cell lines were treated with 1 μM rhBMP-2, resulted in an accumulation of cells in the G1 phase from 41.46 ± 2.23% to 42.95 ± 2.08% (10 nM, p = 0.30), 43.62 ± 2.08% (250 nM, p = 0.23), 44.62 ± 1.33% (500 nM, p = 0.11), and 48.09 ± 1.92% (1000 nM, p = 0.02) and from 39.45 ± 2.67% to 42.33 ± 2.57% (10 nM, p = 0.23), 42.71 ± 2.66% (250 nM, p = 0.21), 45.01 ± 2.61% (500 nM, p = 0.09), and 46.10 ± 2.07% (1000 nM, p = 0.04) at 24 h. Significant differences in the G1 phase cell cycle were only observed after treatment with 1 μM rhBMP-2 compared to the controls in the TE-8 and TE-12 cell lines. Significant differences in the G1 cell cycle were only observed after the treatment with 1 μM of rhBMP-2 compared to the control (Fig. 2B). The other doses of rhBMP-2 did not exhibit any significant changes in the G1 cell cycle distribution, although they had an increased trend towards a dose-dependent manner. Both cell lines treated with 1 μM rhBMP-2 showed not only a decrease in G1 phase-related protein expression, such as cyclin D1, cyclin-dependent kinase (CDK) 4, and CDK 6, but also an increase in expression of p-Smad 1/5/8, p53, and p21 (Fig. 2B,C). These results suggest that comprehensive regulation of p-Smad1/5/8, p53, p21, cyclin D1, CDK 4, and CDK 6 through treatment with rhBMP-2 results in G1 cell cycle arrest.

### Induction of apoptosis in TE-8 and TE-12 cells by rhBMP-2

**Figure 3:**
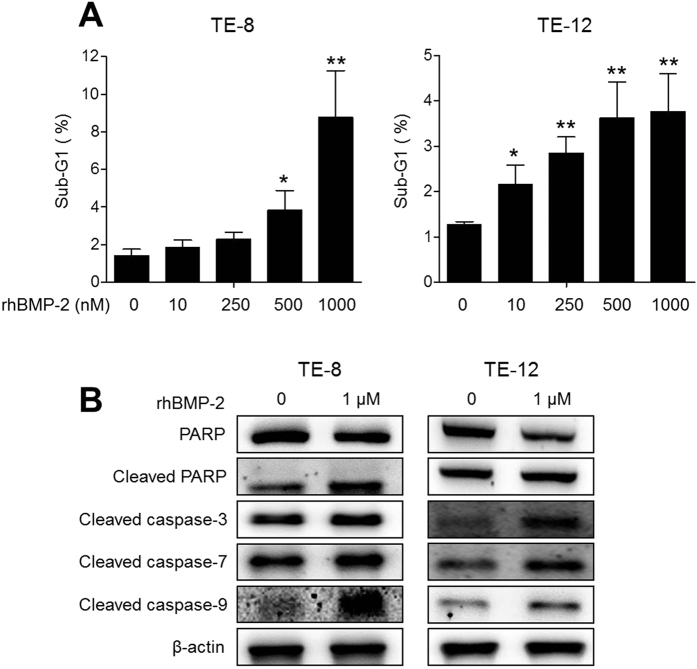
Cell cycle analysis by flow cytometry and the percentage of apoptotic cells in the sub G1 phase. The two cell lines were treated with rhBMP-2, which led to the accumulation of cells in the sub G1 phase in a dose-dependent manner. The percentage of sub G1 cells increased in the TE-8 and TE-12 cell lines after rhBMP-2 treatment (**A**). A Western blot analysis of the association of BMP-2 with apoptosis proteins. Treatment with rhBMP-2 decreased the expression of PARP, whereas cleaved-PARP as well as cleaved-caspase-3, 7, and 9 protein levels increased in the TE-8 and TE-12 cell lines (**B**). The gels have been run under the same experimental conditions. The full-length blots and the cropped blots are presented in Supplementary Figure 3. β**-**actin was used as an internal control.

The two cell lines treated with rhBMP-2 led to an accumulation of cells in the sub G1 phase in a dose-dependent manner at 24 h. The percentages of sub-G1 cells increased from 1.41 ± 0.34% to 1.85 ± 0.38% (10 nM, p = 0.20), 2.27 ± 0.4% (250 nM, p = 0.07), 3.82 ± 1.06% (500 nM, p = 0.03), and 8.77 ± 2.48% (1000 nM, p = 0.009) in the TE-8 cell line and from 1.26 ± 0.06% to 2.15 (±0.43% (10 nM, p = 0.04), 2.84 ± 0.35% (250 nM, p = 0.002), 3.63 ± 0.79% (500 nM, p = 0.01), and 3.76 ± 0.83% (1000 nM, p = 0.01) in the TE-12 cell line after rhBMP-2 treatment (Fig. 3A). Subsequently, treatment with rhBMP-2 decreased the expression of PARP, whereas the cleaved-PARP, cleaved-caspase-3, -7, and, -9 protein levels increased in TE-8 and TE-12 cell lines (Fig. 3B).

### Effect of rhBMP-2 on the Hippo signaling pathway in TE-8 and TE-12 cells

**Figure 4:**
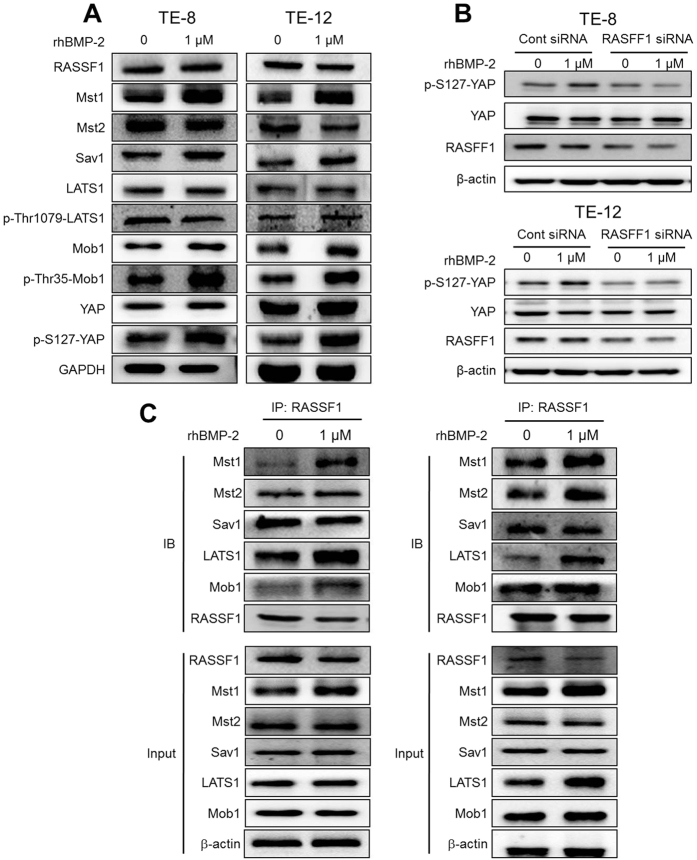
BMP-2 affected Hippo pathway-related proteins in TE-8 and TE-12 cells. Expression of Mst1, p-LATS1, Mob1, Sav1, and p-Mob1 increased markedly after rhBMP-2 treatment in the TE-8 and TE-12 cell lines. In contrast, expression of LATS1 diminished slightly after rhBMP-2 treatment in the TE-8 and TE-12 cell lines. In addition, expression of YAP, a Hippo pathway downstream effector, was not altered, but pYAP expression increased significantly after rhBMP-2 treatment in the TE-8 and TE-12 cell lines (**A**). Western blotting assays for the expression of YAP and p-YAP proteins in RASSF1 siRANA transfected cells treated with or without 1 μM rhBMP-2 for 48 h in TE-8 and TE-12 cell lines (**B**). The interaction between Mst1 and RASSF1 was significantly stimulated by 1 μM rhBMP-2 treatment in the TE-8 and TE-12 cell lines by immunoprecipitation. In addition, rhBMP-2 significantly increased the interactions between LATS1 and Mob-1 with RASSF1 in the TE-8 and TE-12 cell lines (**C**). The gels have been run under the same experimental conditions. The full-length blots and the cropped blots are presented in Supplementary Figure 4. β**-**actin was used as an internal control.

Because Hippo signaling is responsible for organ size and cell proliferation, we examined how rhBMP-2 inhibited growth mediated via the Hippo signaling pathway in the TE-8 and TE-12 cell lines. Expression of Mst1, Mob1, Sav1, and p-Mob1 increased markedly after rhBMP-2 treatment in TE-8 and TE-12 cell lines (Fig. 4A). In contrast, LATS1 expression was not affected and Mst2 expression was diminished slightly after rhBMP-2 treatment in the TE-8 and TE-12 cell lines. In addition, the expression of YAP, a Hippo pathway downstream effector, was not altered, but pYAP expression was significantly increased after rhBMP-2 treatment in the TE-8 and TE-12 cell lines. To test whether the activation of the Hippo pathway induced by rhBMP-2 inhibits esophageal cancer proliferation, we performed a knockdown experiment. Because YAP is a key downstream effector of the Hippo pathway, it was silenced by RNA interference (Supplementary Figure 1). The growth rate of the TE-8 and TE-12 cells transfected with YAP siRNA were significantly inhibited compared with the cells transfected with the empty vector. In addition, silencing of YAP plus rhBMP-2 treatment in TE-8 and TE-12 cell lines resulted in significantly decreased cellular proliferation compared with cells transfected with the empty vector or YAP siRNA, respectively. As rhBMP-2 regulates the Hippo pathway (Fig. 4A), we determined whether rhBMP-2 treatment facilitate RASSF1, an upstream regulator of the Hippo signaling pathway, directly interacting with Mst1/2, LATS1, Sav1, and Mob1. However, rhBMP-2 (1 μM) did not alter RASSF1 expression in the TE-8 and TE-12 cell lines. The interaction between Mst1 and RASSF1 was significantly stimulated by 1 μM rhBMP-2 treatment in the TE-8 and TE-12 cell lines by immunoprecipitation. In addition, rhBMP-2 significantly enhanced the interactions of LATS1 and Mob-1 with RASSF1 in the TE-8 and TE-12 cell lines (Fig. 4C). In addition, we knocked down the expression of RASSF1 in TE-8 and TE-12 cells using siRNA. The expression of p-YAP was increased in TE-8 and TE-12 cells, whereas the expression of YAP was not changed after the treatment with rhBMP-2. The induced p-YAP expression in TE-8 and TE-12 cells by rhBMP-2 was reversed by the RASSF1 knockdown. Taken together, these results suggest that rhBMP-2 inhibits esophageal squamous cell carcinoma via the Hippo pathway (Fig. 4B).

### Effect of rhBMP-2 on Akt in TE-8 and TE-12 cells

**Figure 5:**
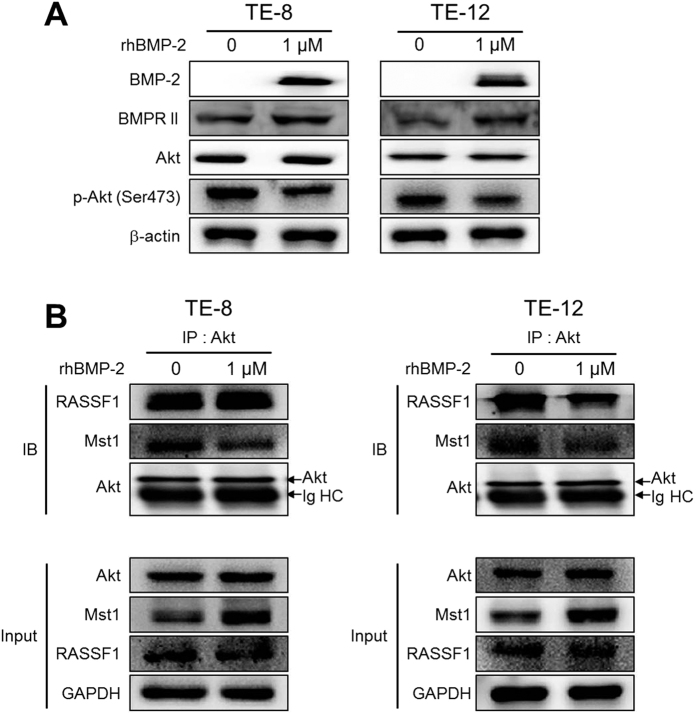
Effect of BMP-2 on Akt signaling pathway-related protein in TE-8 and TE-12 cells. rhBMP-2 significantly increased expression of BMP2 and BMPRII in the TE-8 and TE-12 cell lines. RhBMP-2 drastically inhibited expression of phosphorylated Akt, whereas expression of Akt was not affected by rhBMP-2 in the TE-8 and TE-12 cell lines (**A**). β**-**actin were used as an internal control. The direct interaction between Akt and RASSF1 was significantly diminished by rhBMP-2 treatment in the TE-12 cell line, whereas the interaction between Akt and RASSF1 was not affected in the TE-8 cell line. The interaction between Akt and Mst1 was also significantly inhibited when stimulated by rhBMP-2 in the TE-8 and TE-12 cell lines (**B**). GAPDH were used as an internal control. The gels have been run under the same experimental conditions. The full-length blots and the cropped blots are presented in Supplementary Figure 5.

Because Akt has been linked to cell proliferation and growth, we next examined whether rhBMP-2 could affect the Akt signaling pathway in the TE-8 and TE-12 cell lines. rhBMP-2 (1 μM) significantly increased BMP2 and bone morphogenetic protein receptor II (BMPRII) expression in the TE-8 and TE-12 cell lines. RhBMP-2 drastically inhibited expression of phosphorylated Akt, whereas expression of Akt was not affected by rhBMP-2 in the TE-8 and TE-12 cell lines (Fig. 5A).

To investigate whether rhBMP-2 directly stimulates the interaction between RASSF1 and Akt, we determined whether Akt binds with RASSF1. We found that the direct interaction between Akt and RASSF1 was significantly diminished by rhBMP-2 treatment in the TE-12 cell line, whereas the interaction between Akt and RASSF1 was not affected in the TE-8 cell line. The interaction between Akt and Mst1 was also significantly inhibited when stimulated by rhBMP-2 in the TE-8 and TE-12 cell lines (Fig. 5B).

### Effect of rhBMP2 on subcutaneous tumor formation

**Figure 6:**
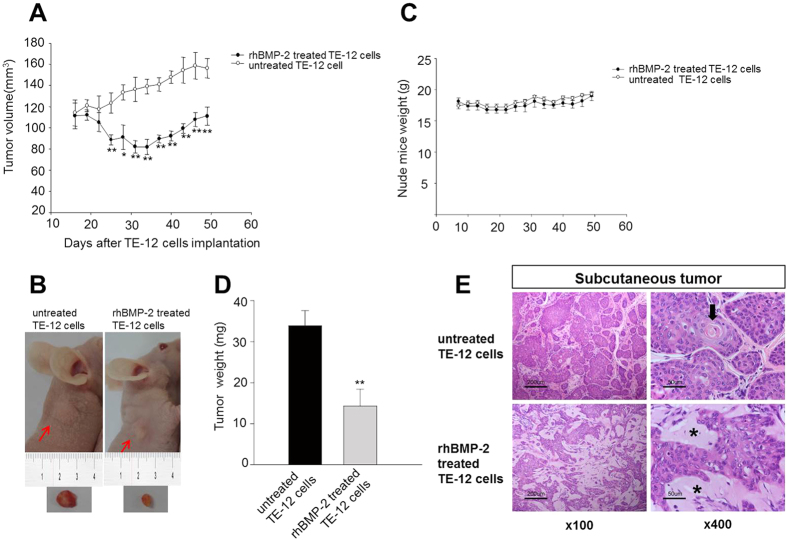
Subcutaneous tumor formation and growth curves of TE-12 cells. The mean size and weight of subcutaneous tumors was lower in the rhBMP-2 treated group than those in the untreated group over time (**A–C**). Compared with rhBMP-2-treated and untreated groups, weight loss of nude mice was not related with rhBMP-2 treatment (**D**). Histological finding of the subcutaneous tumor in the rhBMP-2-untreated and rhBMP-2-treated groups. The stroma between the tumor nests in the rhBMP-2-untreated group was narrow and contained fibroblast and inflammatory cells. Arrow indicates squamous pearl of TE-12 squamous cell carcinoma. In contrast, the intervening stroma between the tumor cell nests in the rhBMP-2 treated group was wide and the stroma was hypo-cellular, amorphous, and basophilic (asterisk) (**E**). Data are mean ± standard error, ^*^P < 0.05.

Based on our *in vitro* study, we designed further experiments to investigate the effects of rhBMP-2 on xenograft implanted human esophageal tumors in nude mice. Subcutaneous tumors were established by injecting TE-12 cells (5 × 10^6^ cells with or without co-injecting rhBMP-2 into subcutaneous tissue in the flank area of nude mice). Mean subcutaneous tumor size was lower in the rhBMP-2 treated group than that in the untreated group over time (Fig. 6A–C). No significant change in mean animal weight was observed between the untreated and rhBMP-2 treated groups, indicating that there was no toxicity to the nude mice (Fig. 6D). No difference in the histologic findings of TE-12 squamous cell carcinoma nest was observed between the rhBMP-2-untreated and the rhBMP-2-treated groups. The tumor formed keratin pearls and showed intercellular bridges in both groups, which are characteristic findings of squamous cell carcinoma. However, the stroma between the tumor cell nests was different. The stroma was narrow and contains fibroblast and inflammatory cells in the rhBMP-2-untreated group, whereas the stroma in the rhBMP-2 treated group was wide, hypocellular, amorphous, and basophilic (Fig. 6E).

### Femur implantation and radiographic analysis

**Figure 7:**
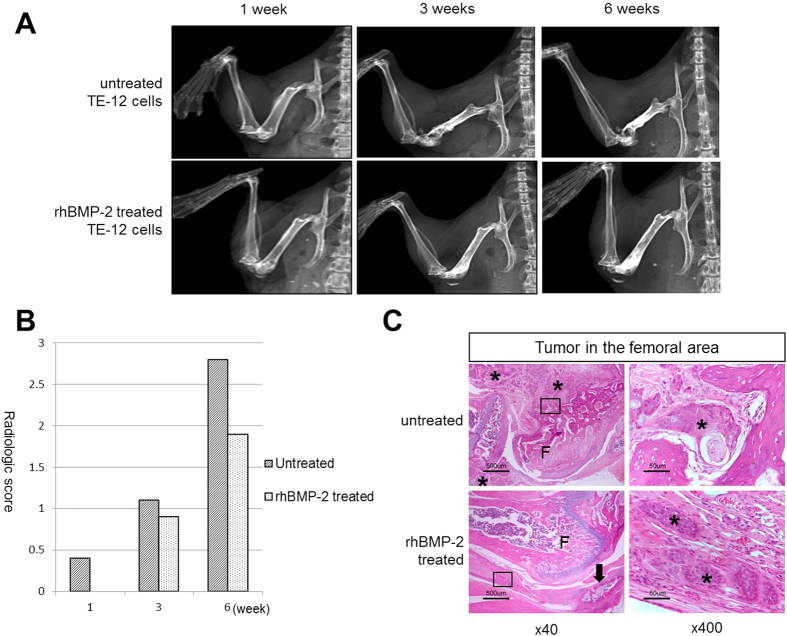
Radiographic analysis. Radiographs show destructive lesions in the untreated group and both osteolytic and osteoblastic lesions in the rhBMP-2 treated groups (**A**). The mean radiological score of the untreated group was consistently greater than that of the cells treated with rhBMP2 group, but the difference reached statistical significance only at 6 weeks (**B**, ^*^P < 0.05). (**C**) Histologic finding of the femoral- and peri-femoral area implanted with TE-12 cells in the rhBMP-2-untreated and rhBMP-2-treated groups. The TE-12 squamous cell carcinoma in the rhBMP-2-untreated group formed a large mass in the femoral area and destructively infiltrated the femur and adjacent soft tissue. In contrast, small amounts of tumor cells were identifiable and the femur was relatively intact in the rhBMP-2 treated group. Arrow indicates new bone formation in the soft tissue around the distal femur of the rhBMP-2 treated group. The black-lined boxes indicate right sided high-power field areas. The asterisks indicate tumor cells and ‘F’ indicates femur.

Radiographs were obtained at 1, 3, and 6 weeks after injection. Two independent reviewers who were blinded to the treatment groups analyzed the radiographs for the presence of osteoblastic and osteolytic lesions. The radiographs showed destructive lesions in the untreated group and both osteolytic and osteoblastic lesions in the rhBMP-2 treated groups (Fig. 7A). The mean radiological score in the untreated group was consistently greater than that of the cells in the rhBMP2 group, but this difference reached significance only at 6 weeks (Fig. 7B).

The TE-12 squamous cell carcinoma formed a large tumor mass in the femoral area and destructively infiltrated the femur and adjacent soft tissue in the rhBMP-2-untreated group. In contrast, fewer tumor nests and a relatively intact femur were observed in the rhBMP-2 treated group compared with those in the rhBMP-2 untreated group. In addition, new bone formation was found in the rhBMP-2 treated group (Fig. 7C).

## Discussion

**Figure 8:**
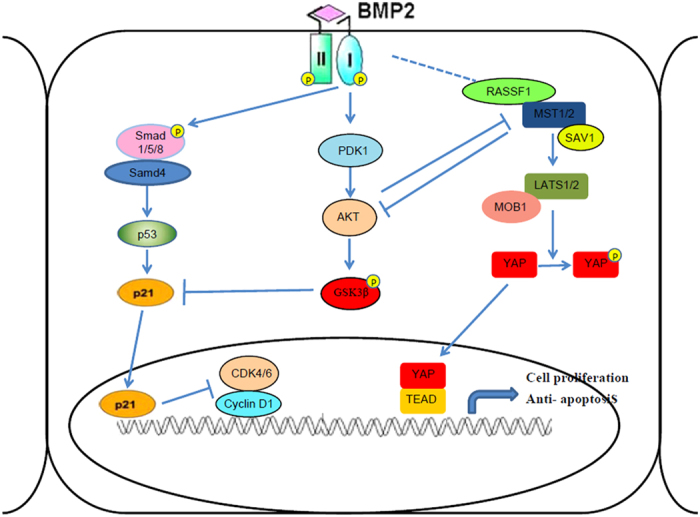
Schematic representation of BMP-2 signaling pathway negatively regulating proliferation of esophageal cancer cells via induction of p21 through the SMAD pathway and inhibit YAP translocation to the nuclear through Hippo signaling pathway.

The main focus of this study was to assess the regulation of cell growth in esophageal cancer cells (*in vitro* and *in vivo*) and the possible usefulness of rhBMP-2 in patients with esophageal cancer because rhBMP-2 is often used in tissue engineering for spine defect regeneration. Our observations show that rhBMP-2 inhibited esophageal cancer cell growth by activating Hippo pathway components as well as YAP function (Fig. 8). Furthermore, we showed that rhBMP-2 strongly inhibited esophageal human tumor cells in xenografted nude mice. These results indicate that rhBMP-2 could be used for spinal tissue engineering and reconstructive spine surgery for the defect area in patients with esophageal cancer.

BMP-2 is a multi-functional growth factor with several effects on cell growth and development activity^19^. Many researchers have reported the biological effects of BMPs on cancer cells. For example, inhibited cell growth by rhBMP-2 treatment has been described previously in breast, gastric, and colon cancer cells^8^,^20^,^21^,^22^. In contrast, BMP-2 stimulates cell proliferation in lung and prostate cancer cells^19^,^23^,^24^. A recent study revealed that exposure of oral squamous cell carcinoma to rhBMP-2 does not stimulate proliferation of cells or increase tumor volume^19^. In the present study, the MTT assay showed that rhBMP-2 significantly suppressed proliferation of esophageal cancer cells. In addition, the number of colonies was significantly diminished by rhBMP-2 treatment of esophageal cancer cells. We further investigated how rhBMP-2 mediates the antiproliferative effect in esophageal cancer cells. We found that rhBMP-2 induced the expression of apoptotic proteins (cleaved caspase-3, -7, -9, and PARP). Consistent with these effects, rhBMP-2 suppressed activation of the G1 cell cycle proteins (cyclin D1, CDK4, and CDK6). These results of suppressed G1 cell cycle proteins by rhBMP-2 are in agreement with our previous research on breast cancer cells^8^.

Although BMP-2 inhibits the proliferation of many cancer cells including breast, gastric, and colon cancer cells, the specific mechanisms of rhBMP-2 in esophageal squamous cancer cell death *in vitro* or *in vivo* have never been clearly elucidated. Hence, we next examined whether rhBMP-2 regulates cell death mediated through the Hippo signaling pathway in esophageal squamous cancer cells. The Hippo pathway has been of great interest to researchers because the downstream effector of the Hippo pathway, YAP, has an important role in cancer development and progression^12^,^25^. In addition, dysregulation of the Hippo pathway has been implicated in many types of cancers^25^,^26^. For example, decreased expressions of Mst1/2 and LATS are found in gastric cancer, and overexpressed YAP proteins are strongly related to a poor prognosis of patient survival^27^. In the present study, we found for the first time that rhBMP-2 significantly increased expression of the Hippo pathway components (Mst1, Sav1, Mob1, and pMob1). Phosphorylated YAP, an inactivate form of YAP, was also significantly stimulated by rhBMP-2 treatment. In addition, silencing of YAP significantly inhibited the growth rate of TE-8 and TE-12 cells. Moreover, the silencing of YAP plus rhBMP-2 treatment in TE-8 and TE-12 cells resulted in significantly decreased cellular proliferation compared with cells transfected with an empty vector or with YAP siRNA, respectively. These results suggest that rhBMP-2 suppresses the proliferation of esophageal cancer cells mediated through the Hippo signaling pathway.

Because the ras association domain family 1(RASSF1) regulates both the cell cycle and apoptosis, it is thought to function as a tumor suppressor. RASSF1 is frequently inactivated in lung, breast, gastric, and other cancer cells and suppresses tumor cell growth *in vivo* and *in vitro*^15^,^28^,^29^,^30^,^31^,^32^,^33^. In addition, members of the RASSF family of proteins interact with Mst1 kinase^32^,^33^,^34^. Oh *et al*. also described that overexpression of RASSF1 increased Mst 1 kinase activity and promotes apoptosis^32^. In the present study, because rhBMP-2 increases Mst1 activation, we further investigated whether the RASSF1 protein was associated with Mst1 when stimulated by rhBMP-2. Our results showed that the core components of the Hippo pathway (Mst1/2, LATS1, and Mob1) and the RASSF1 protein coimmunoprecipitated and that their binding was significantly enhanced by rhBMP-2 treatment in both esophageal cancer cell lines. In addition, the induced expression of p-YAP in TE-8 and TE-12 cells by rhBMP-2 was inhibited by the RASSF1 silencing. These results suggest that rhBMP-2 may induce RASSF1, which interacts with Mst1, and promotes apoptosis in esophageal cancer cells.

Our data demonstrate that rhBMP-2 can be used as an antineoplastic agent in this *in vivo* model of xenografted esophageal cancer cells. Tumor masses treated with rhBMP-2 significantly decrease in size with decreased skeletal invasion. This model showed both similar and opposite effects as our previous studies^8^,^35^. In the lung cancer model, inhibited BMP2 activity resulted in reduced tumor growth, whereas in this model of metastatic esophageal cancer, increased BMP2 activity reduced tumor growth. Concern of the cancer risk from rhBMP-2 following spinal fusion is increasing. One study^36^ reported that the use of rhBMP-2 during spinal fusion may increase the number of cancer cases, but a York University^37^ report found no significant difference in cancer prevalence between rhBMP2 use and no use. Our findings and previous reports highlight the critical need to individualize antineoplastic therapy based on the response to growth factors such as BMPs.

## Conclusion

Our results suggest that rhBMP-2 inhibited proliferation of human esophageal cancer cells rather than activated them which was mediated by activating the Hippo signaling pathway. Therefore, rhBMP-2 can be used as an antineoplastic agent in this *in vivo* model of xenografted esophageal cancer cells.

## Methods

### Reagents and cell lines

Recombinant human BMP-2 was purchased from DaeWoong Pharmaceutical (Seoul, South Korea). Cell cycle related protein antibodies (cyclin D1, CDK4, p21, p53, p-Smad, and CDK6) and Hippo signaling pathway related protein antibodies (Mst1, Mst2, Sav1, LATS1, p-LATS1, Mob1, p-Mob1, YAP, and p-YAP) were obtained from Cell Signaling Technology (Danvers, MA, USA). The BMP-2 antibody was obtained from Abcam (Cambridge, UK) and BMPR II, RASSF1, Akt, p-Akt, TP63, and β-actin antibodies were from Santa Cruz Biotechnology (Santa Cruz, CA, USA). RASSF1 small interfering RNA (siRNA), YAP siRNA, or control siRNA were purchased from Santa Cruz biotechnology (Dallas, TX, USA). The human TE-8 and TE-12 esophageal cancer cell lines were obtained from Dr. Izzo (Unversity of Texas M.D. Anderson Cancer Center, Houston, TX, USA). DMEM-F12 medium (Gibco, Grand Island, NY, USA) with 10% fetal bovine serum (Gibco), 100 mg/ml streptomycin, and 100 IU/ml penicillin (Gibco) was used for cell medium. Cells were maintained under standard conditions at 37 °C in a 5% CO_2_ humidified atmosphere.

### Cell proliferation assay

The MTT assay was performed to determine cell proliferation as described previously by our group^8^. Briefly, TE-8 and TE-12 cells were seeded with 10^4^ cells/well plates and were allowed to adhere. Various concentrations of rhBMP-2 were added to the TE-8 and TE-12 cells after a 24 h incubation under standard conditions. Optical density was determined at a wavelength of 570 nm using the Epoch microplate spectrophotometer (Biotek Instrument, Inc., Winooski, VT, USA).

### Cell cycle analysis

TE-8 and TE-12 cells were cultured in complete medium overnight and then treated with rhBMP-2 in a dose dependent manner 24 h. The cells were washed twice with ice-cold PBS, followed with fixed in cold 70% ethanol overnight. The next day, the cells were washed with PBS to remove the ethanol. Subsequently, RNase was added into resuspend cell solution, incubate in CO_2_ incubator 37 °C for 15 min. Finally, PI staining was performed. The cell cycle populations of TE-8 and TE-12 cells lines were measured with a FACstar flow cytometer (Becton Dickinson, San Jose, CA, USA) and analyzed with Becton Dickinson software (LYSIS II, CELL FIT).

### Western blot analysis

Cells were seeded in 10 cm dishes at a density of 1 × 10^6^ cells per dish. TE-8 and TE-12 cells were exposed to various concentrations of rhBMP-2. Then, the cells were washed with PBS and harvested. Cell pellets were lysed in ice-cold PRO-PREP^TM^ (Intron Biotechnology, Deajeon, Korea). The proteins were separated on 10% sodium dodecyl sulfate-polyacrylamide gel electrophoresis (SDS-PAGE) and transferred to PVDF membranes (GE Healthcare Life Sciences, Buckinghamshire, UK). The blot was probed antibodies against BMP-2, BMPR II, cyclin D1, CDK4, CDK6, p21, p53, p-Smad1/5/8, PARP, cleaved PARP, cleaved caspase-7, cleaved caspase-9, Akt, p-Akt, Mst1, Mst2, Sav1, Mob1, p-Mob1, LATS1, p-LATS1, YAP, p-YAP, and β-catenin overnight followed by further incubation with secondary antibody-horseradish peroxidase. Immunoreactivity was detected using by Fusion Fx7 (Vilber, France).

### Immunoprecipitation

Cells were lysed in 20 mM Tris HCl (pH 8), 10% glycerol, 137 mM NaCl, 1% Nonidet P-40, and 2 mM EDTA. After centrifugation at 12,000 rpm for 10 min at 4 °C, protein concentration was quantified (1 mg/ml). Immunocomplexes were collected after incubation with Protein A-Sepharose beads (Sigma-Aldrich, St. Louis, MO, USA). A mouse monoclonal RASSF1 was used to immunoprecipitate Mst1, Mst2, Sav1, LATS1, and Mob1. Immunoprecipitates were washed three times with lysis buffer. Cell lysates were separated by SDS-PAGE.

### *In vivo* animal experiment

Five-week-old female nude mice (BALB/c nu/nu, n = 20) were purchased from Orient Bio company (Deajeon, Korea) and used for the experiment. The mice were housed under specific pathogenic free conditions and were allowed to adjust to local conditions for 1 week before cancer cells were injected. Animals were cared for in accordance with the National Institutes of Health Guidelines for Animal Care. All experimental procedures were approved by the Institutional Animal Care and Use Committee at Chonbuk National University (Approved number: CBU 2013-0002). The mice were randomized into two groups, and each group underwent implantation of cells subcutaneously and in to the intra-femoral space following our previous study^8^. Group I animals (n = 10) received TE-12 esophageal cancer cells alone as a control group. Group II animals (n = 10) received TE-12 esophageal cancer cells with rhBMP-2 (10 μg/10 μl).

### Subcutaneous implantation of TE-12 cells and direct measurement of tumor size

TE-12 cells were implanted subcutaneously as described in our previous study. Briefly, 5 × 10^6^ TE-12 cells were injected subcutaneously into each mouse. RhBMP-2 was used to pretreat the TE-12 cells at a dose of 10 μg/10 μL (2 mg/kg). The RhBMP-treated TE-12 cells were prepared in 20 μL PBS with 20 μL Matrigel (11.2 mg/mL, BD Biosciences, San Diego, CA, USA) for each mouse. TE-12 cells alone and a mixture of TE-12 cells with rhBMP-2 (rhBMP-2 was directly added to the TE-12 cells before the injection) were injected subcutaneously into the backs of nude mice. Approximately 2 weeks following implantation, the tumor size reached approximately 100 mm^3^, and we started to measure the tumor sizes. The size of the tumors was measured once every 3 days and calculated according to the formula as tumor volume = (L × W^2^) × 0.5 using digital calipers. These measurements were repeated by three readers blinded to the treatment groups.

### Femur implantation and radiographic analysis

Implantation of cancer cells suspended in Matrigel within the femur was performed as described previously^8^,^35^. Mice were anesthetized with isoflurane, then maintained via an isoflurane face mask. A total of 5 × 10^6^ cancer cells in 15 μL PBS with or without rhBMP2 was injected into the distal femur cavity with a 27 gauge needle. Radiographs were obtained after injection 1 weeks, 3 weeks and 6 weeks respectively. Three independent observers blinded to the treatment groups evaluated the radiographs for the presence of osteoblastic and osteolytic lesions. Radiographs were scored on a scale of 0–3 (0: normal or osteoblastic lesions present; 1: lytic lesions present within the medullary canal only; 2: lytic lesions involving one cortex; 3: lytic lesions involving both cortices).

### Histological methods

Tissues from the femoral and subcutaneous tumor masses were fixed in 10% neutral-buffered formalin. After fixation, the femoral samples were decalcified in rapid decalcifying solution (Calci-Clear Rapid, National Diagnostics, Atlanta, GA, USA) for 12 h and then embedded in paraffin. Tissues were sectioned longitudinally at a thickness of 4 μM and stained with hematoxylin and eosin for light microscopic analysis.

### Small interfering RNA suppression of gene expression

TE-8 and TE-12 cells (2 × 105 cells/well) were cultured in a six-well tissue plate were transiently transfected with 100 pM of RASSF1 small interfering RNA (siRNA), YAP siRNA, or control siRNA (Santa Cruz biotechnology) according to the manufacturer’s instructions. At 24 h after transfection, the cells were used for further treatment.

### Statistical analysis

The statistical analysis was performed using GraphPad Prism Software. Data are presented as mean with standard error. A one way ANOVA followed by a Student’s *t*-test was used to determine if the results were statistically significant. A P value < 0.05 was considered statistically significant. Data were reported as biological replicates, with the number of technical replicates indicated in the figure legends.

## Additional Information

**How to cite this article**: Kim, S. M. *et al*. RhBMP-2 Activates Hippo Signaling through RASSF1 in Esophageal Cancer Cells. *Sci. Rep.* **6**, 26821; doi: 10.1038/srep26821 (2016).

## Supplementary Material

Supplementary Information
