# Supplementary material for: RhBMP-2 Activates Hippo Signaling through RASSF1 in Esophageal Cancer Cells
Source: Sci Rep. 2016 May 27;6:26821. doi: 10.1038/srep26821 (PMC4882600; doi:10.1038/srep26821)

RhBMP-2 Activates Hippo Signaling through RASSF1 in Esophageal Cancer Cells

Soo Mi Kim PhD [1, a,*], Shuai Ye MS [2, a], So-Young Rah PhD[3], Byung Hyun Park PhD[3], Hongen Wang MS[4], Jung-Ryul Kim MD[2], Seung Ho Kim MD[2], Kyu Yun Jang MD[5], Kwang Bok Lee MD, PhD[2,*]

Department of Physiology[1], Orthopedic Surgery[2], Biochemistry[3], Gastroenterology[4], and Pathology[5], Institute for Medical Sciences, Research Institute of Clinical Medicine of Chonbuk National University-Biomedical Research Institute of Chonbuk National University Hospital Chonbuk National University Medical School, Jeon Ju, 561-180, Republic of Korea

[a,*] These authors contributed equally to this work.

Soo Mi Kim [1, a, *]
Email: soomikim@jbnu.ac.kr
Shuai Ye [2, a,]
Email: yeshuai1986@163.com
So-Young Rah[3]
Email: syrah1004@hanmail.net
Byung Hyun Park[3]
Email: bhpark@jbnu.ac.kr
Hongen Wang[4]
Email: whe328@163.com
Jung-Ryul Kim[2]
Email: jrkeem@jbnu.ac.kr
Seung Ho Kim[2]
sh1203kim@hanmail.net
Kyu Yun Jang[5]
kyjang@jbnu.ac.kr
Kwang Bok Lee[2, *]
Email: osdr2815@naver.com

Supplementary Figure 1

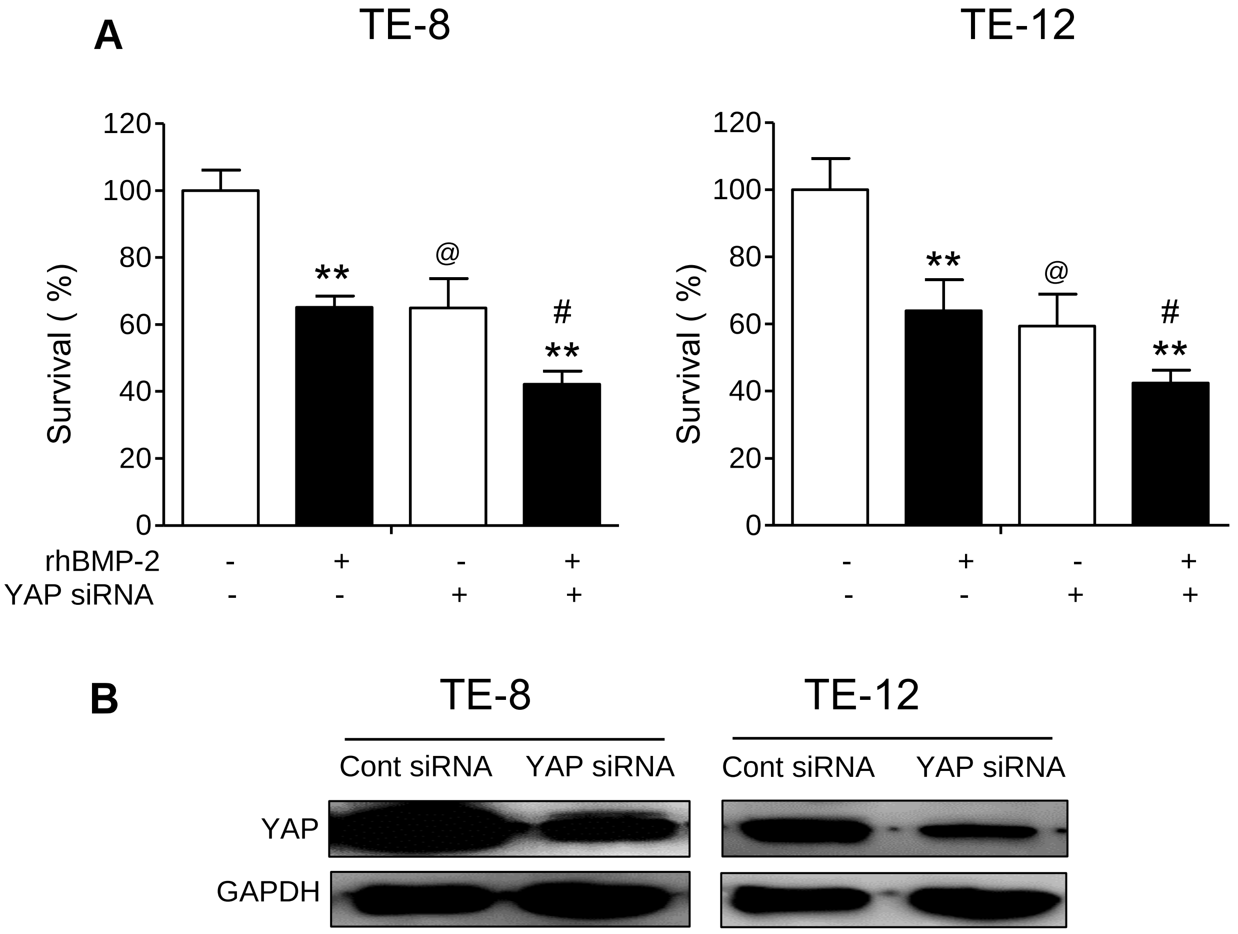

Supplementary Figure 1. A cell survival assay in TE-8 and TE-12 cells treated with rhBMP-2 for 48 h after transient transfection with siRNA against YAP (A). RhBMP-2-inhibited cellular proliferation and the viability of TE-8 and TE-12 cells were more significantly decreased by rhBMP-2 treatment with YAP siRNA transfection compared to rhBMP-2 treatment alone. Each value represents the mean ± standard error of at least three independent experiments with triplicate plates, $^{**}P < 0.01$; control vs. rhBMP-2 treated cells, $^{\#}P < 0.05$; rhBMP-2-treated cells vs. rhBMP-2 plus YAP siRNA transfected cells, $^{@}P < 0.05$; control vs. YAP siRNA transfected cells. Western blotting assays for the expression of YAP in control and

YAP siRANA transfected cells for 48 h in TE-8 and TE-12 cell lines (B). GAPDH were used as an internal control.

Supplementary Figure 2

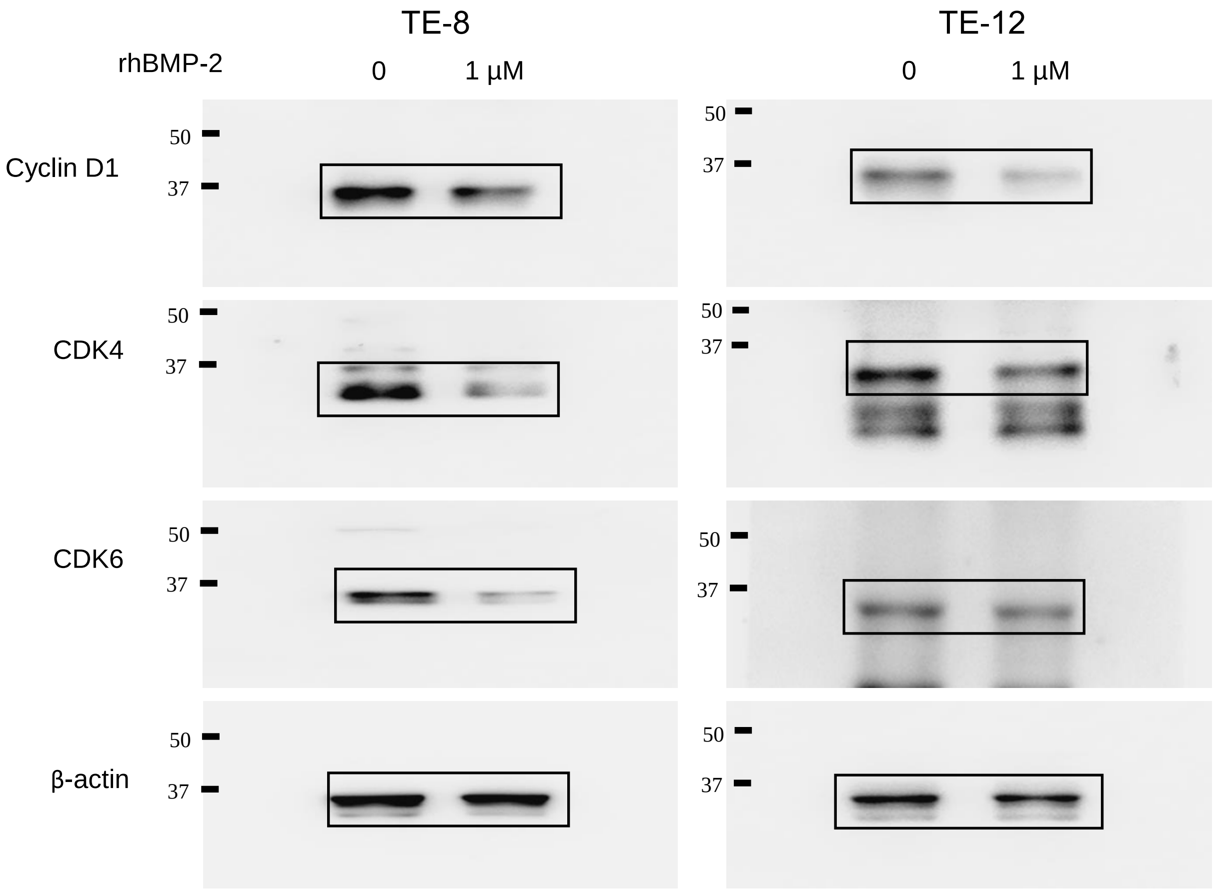

Supplementary Figure 2

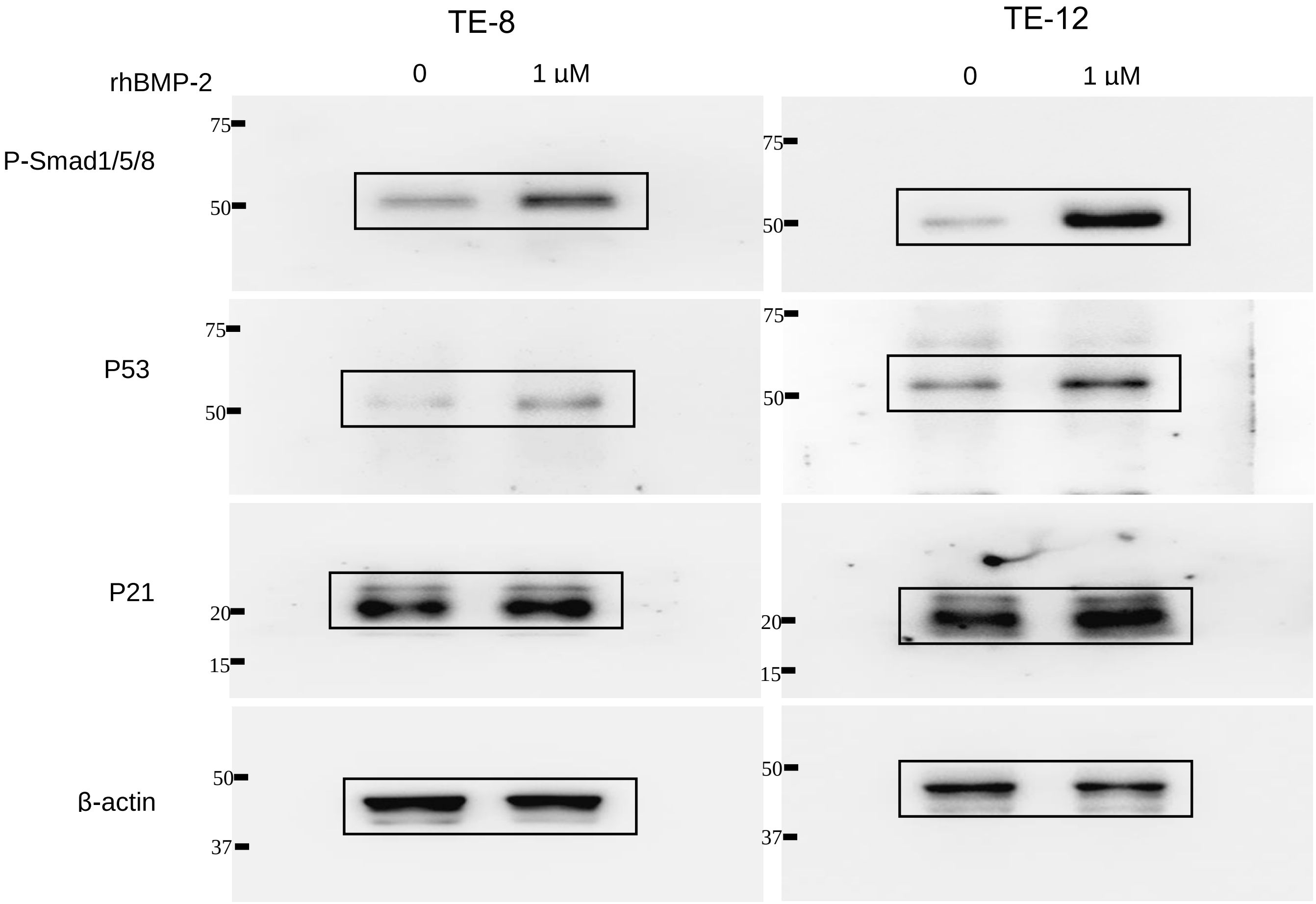

Supplementary Figure 3

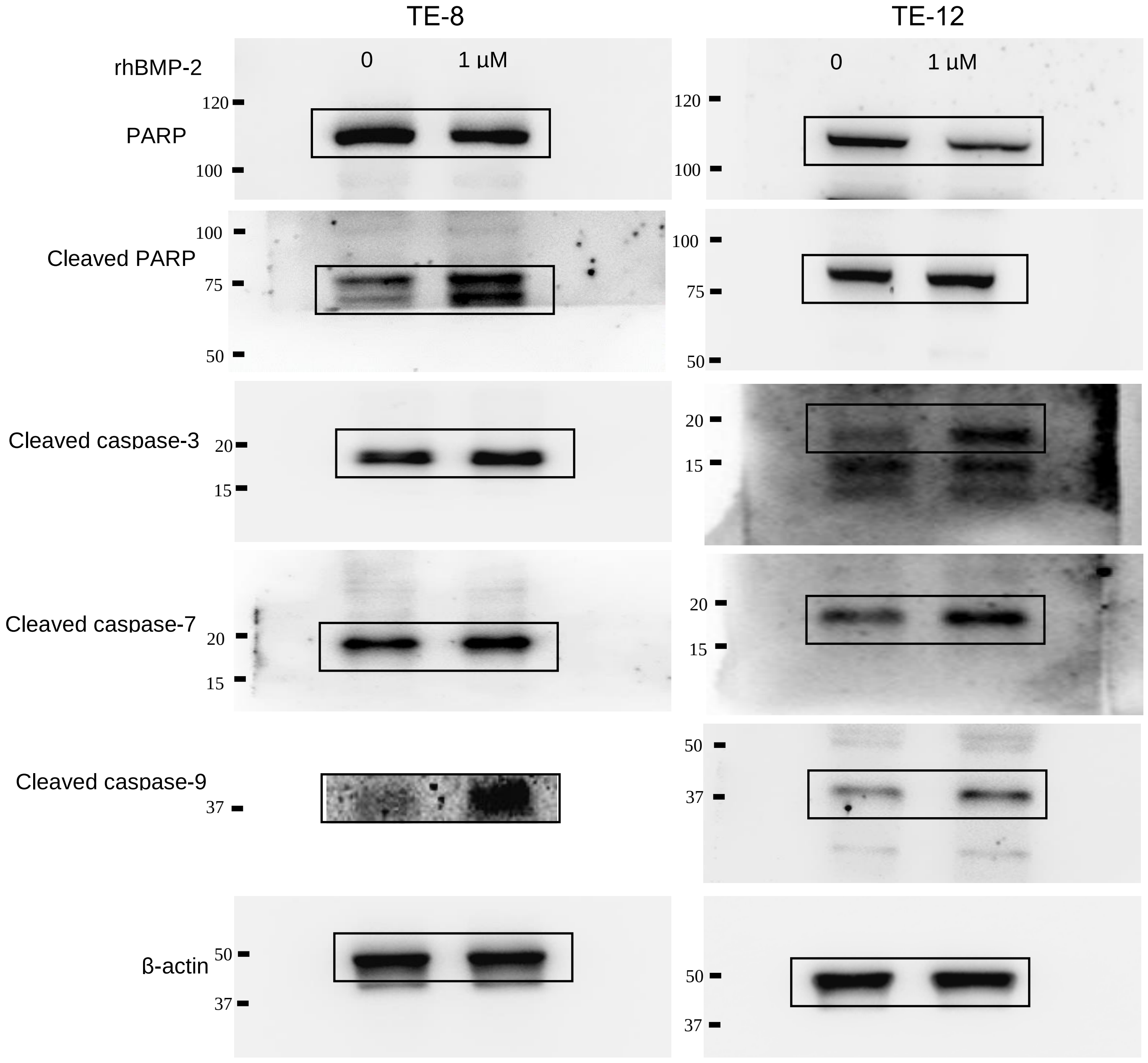

Supplementary Figure 4

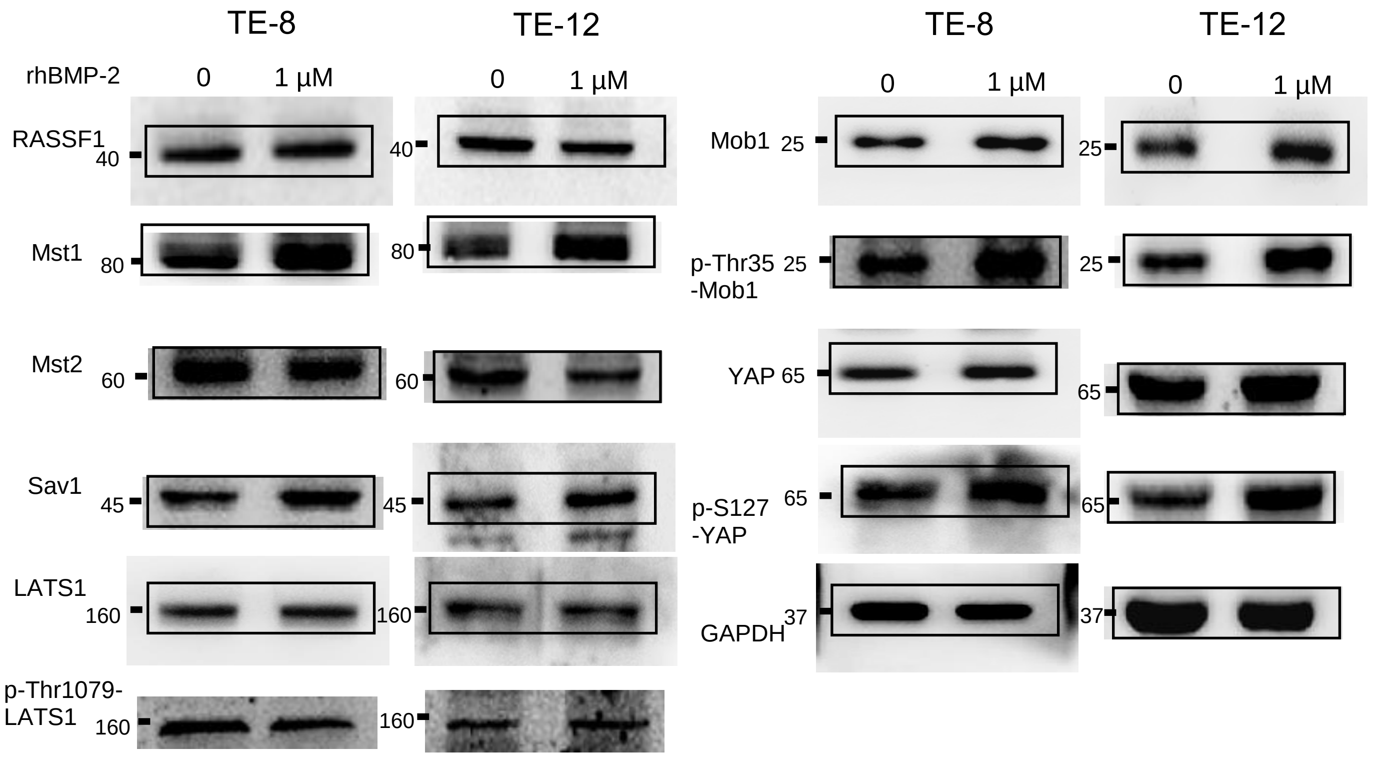

Supplementary Figure 4

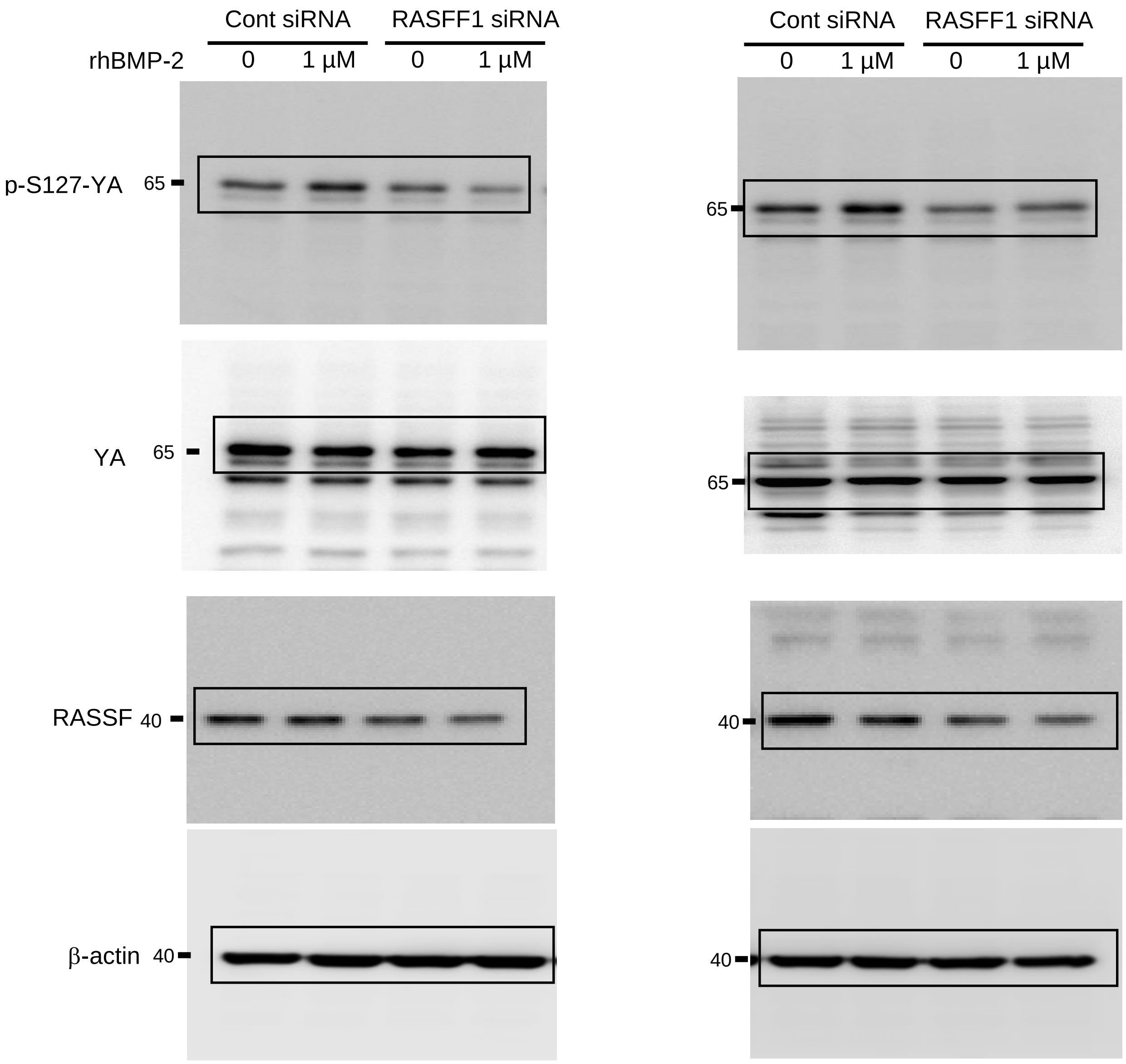

Supplementary Figure 4

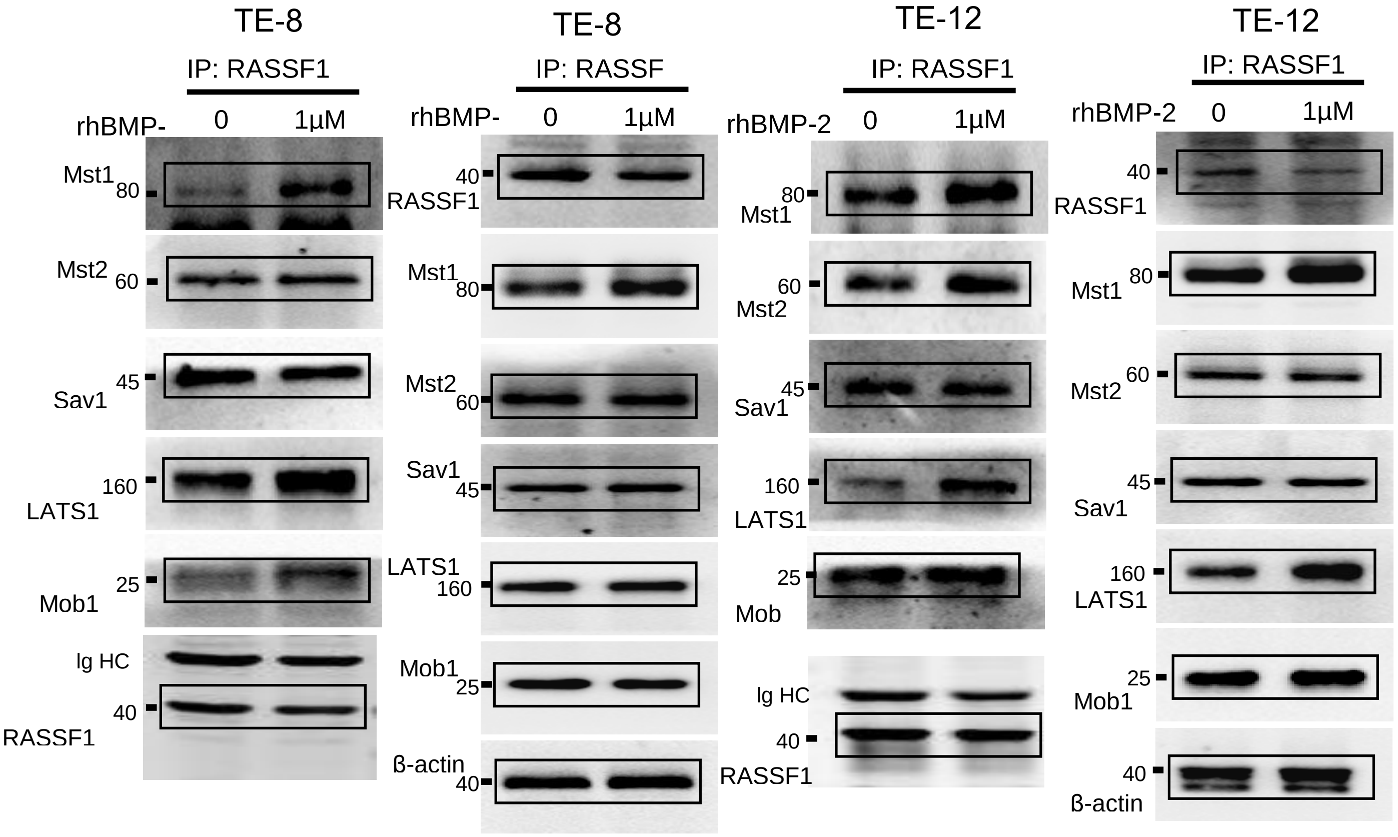

Supplementary Figure 5

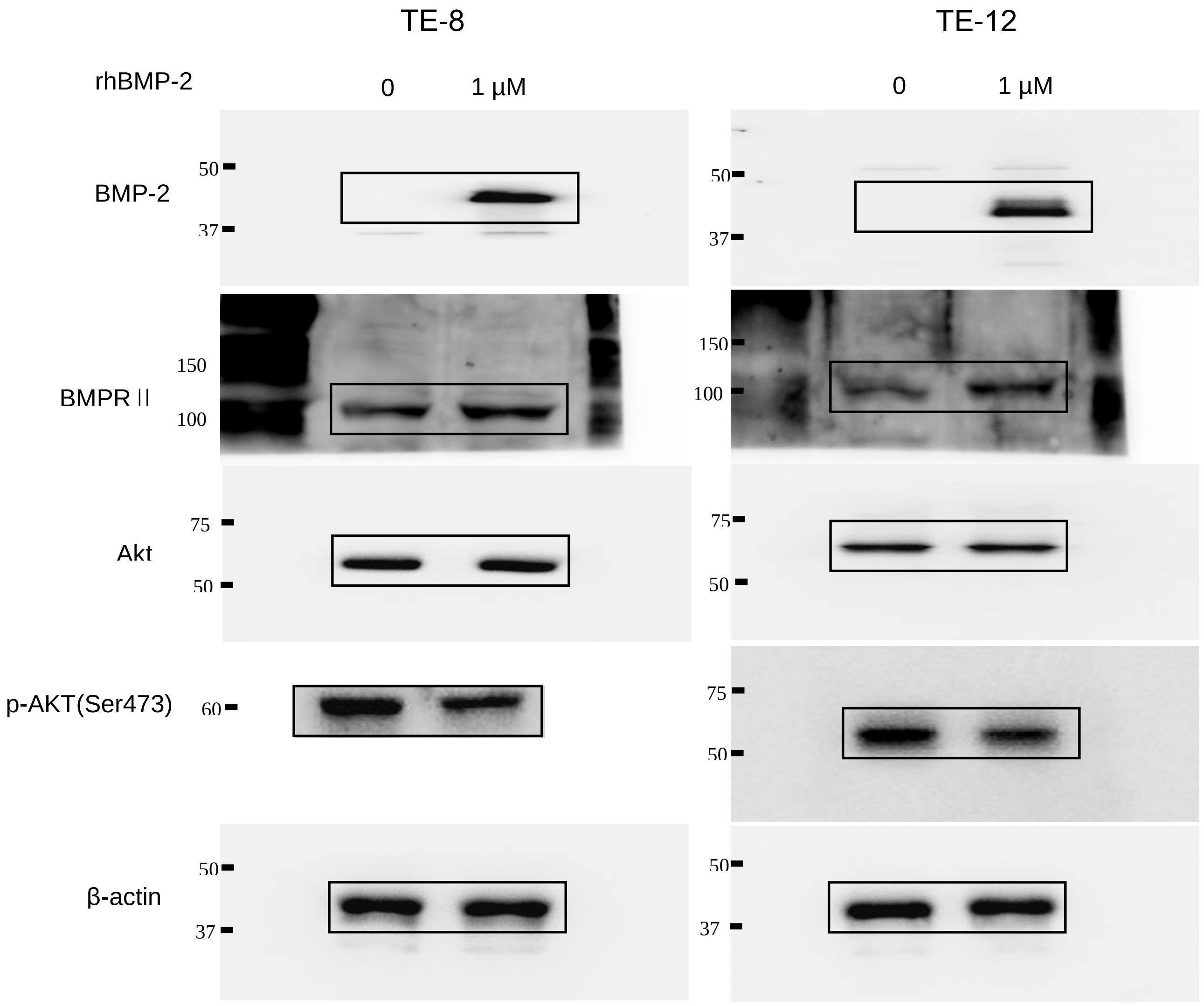

Supplementary Figure 5

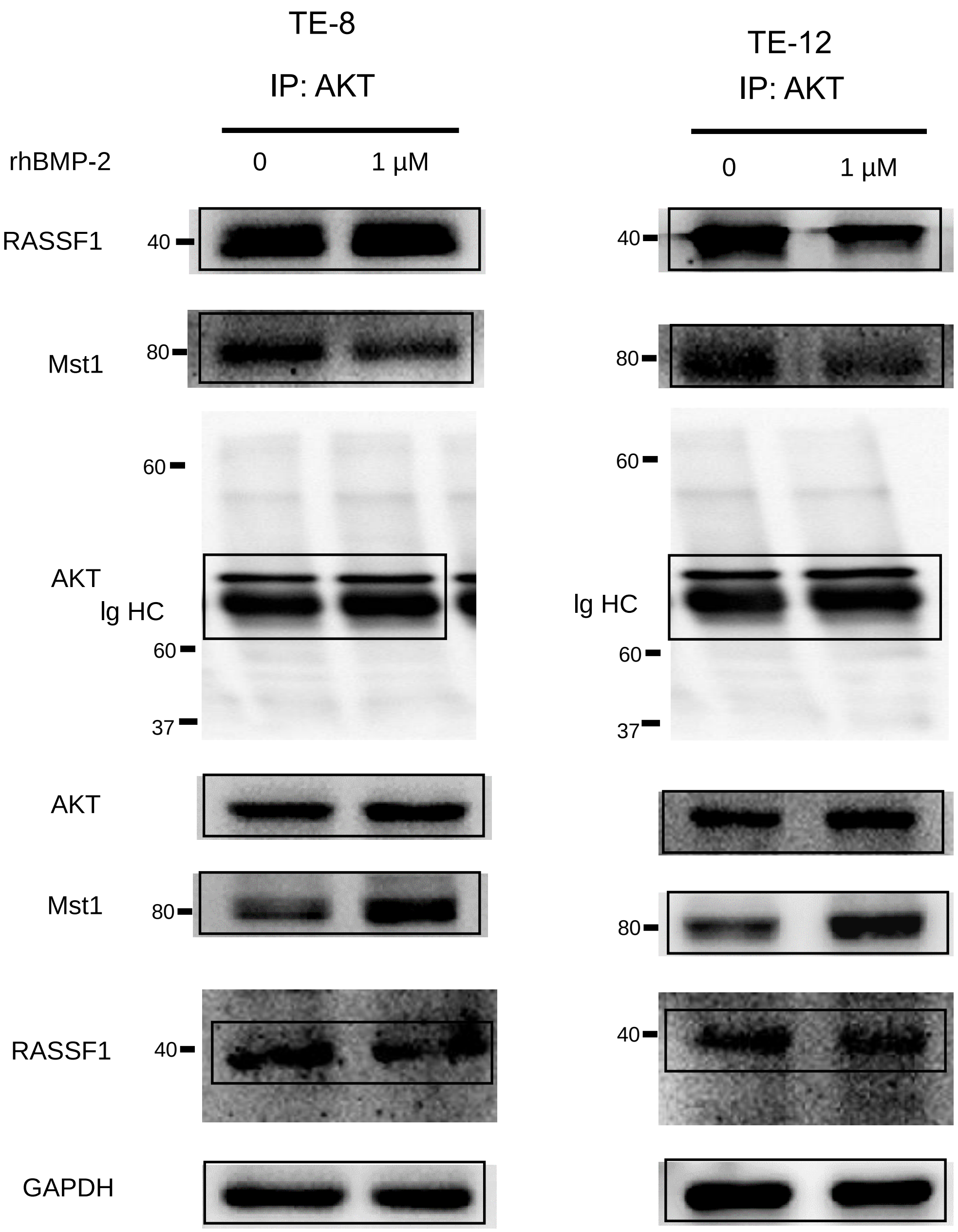

Supplement: Supplementary Information [file srep26821-s1.pdf]
